# Home delivery service of low protein foods in inherited metabolic disorders: Does it help?

**DOI:** 10.1016/j.ymgmr.2019.100466

**Published:** 2019-03-22

**Authors:** A. MacDonald, A. Pinto, S. Evans, C. Ashmore, J. MacDonald, A. Daly

**Affiliations:** Birmingham Women’s and Children’s Hospital, Birmingham, UK

**Keywords:** Low protein foods, Home delivery service, Prescriptions, IMD

## Abstract

**Background:**

In the UK, the customary method of obtaining special low protein (LP) foods was by dispensing through a pharmacist (until 2010) for patients with inherited metabolic disorders (IMD) requiring LP diets. Recently, different home delivery services have been introduced to support patient access of low protein foods, but the effectiveness of these services is unclear.

**Aim:**

A prospective, longitudinal, observational study to examine the effectiveness and safety of patient home delivery services for LP foods over 12 months in IMD patients requiring a LP diet.

**Methods:**

IMD patients/caregivers had the choice of 2 home delivery services (Homeward® and Vitaflo at Home®) as well as access to primary care pharmacy services. Both home delivery services provided a limited range of LP foods. Over a 12-month period, a member of the IMD dietetic team conducted 4 home visits to IMD patients on LP diets using home delivery services for low protein foods. At each visit, caregivers completed a questionnaire consisting of 20 multiple choice and open questions about their prescription experience with special LP foods. The researchers also completed stock checks, assessed ‘use by dates’ and adequacy of home storage for LP foods.

**Results:**

In total, 58 patients participated in this study. Over 12 months, 95% (*n* = 55/58) of caregivers used their local pharmacy, 93% (*n* = 54/58) Homeward® and 78% (*n* = 45/58) Vitaflo at Home® to access LP foods. Two home delivery services were used by 41 (71%) caregivers and the remaining 17 (29%) only used one of the home delivery service companies. Each patient only stored a median of 6 (range 0–22) different LP foods at home. Overall, 45% (*n* = 26/58) of caregivers reported problems with their GP prescriptions. 30% (*n* = 16/53) of caregivers received at least one incorrect prescription when using their pharmacy (e.g. gluten-free foods instead of LP, incorrect product or incorrect product amount), 6% errors (*n* = 3/53) with Homeward® and 2% (*n* = 1/48) with Vitaflo at Home®. 49% (*n* = 26/53) of caregivers said they experienced delayed receipt of LP foods from their pharmacy, compared with 11% (*n* = 6/55) from Homeward® and 8% (*n* = 4/48) Vitaflo at Home®.

**Conclusions:**

Although home delivery services for special LP foods are associated with less errors and delay compared with pharmacies, inaccuracies and inefficiencies still occur and the overall system is complex. We suggest a new, simpler, less fragmented system whereby metabolic dietitians prescribe LP foods. This is likely to result in less burden on NHS resources and ensure a better treatment delivered to IMD patients.

## 1 Introduction

Children and adults with inherited disorders of amino acid or protein metabolism such as phenylketonuria (PKU) or tyrosinaemia require low protein (LP) diets, as part of their treatment in order to prevent accumulation of offending amino acids or their metabolites [1]. This necessitates a severe restriction of high protein foods including regular bread, pasta, cereals, cakes and biscuits. There are few foods that patients can eat in unlimited quantities except for most fruits and some vegetables. Consequently, the diet requires supplementation with special LP foods and amino acid supplements free of ‘offending’ precursor amino acids. Special LP foods are regulated by the European legislation ‘Foods for Special Medical Purposes’ (Commission Directive 1999/21/EC of 25 March 1999; amended in Directive 2006/141/EC) [2]. In the UK, 141 LP products are approved by the Advisory Committee on Borderline Substances (ACBS) and prescribed via a *FP10* prescription (this is a form listing items to be prescribed which can be issued and signed by a general practitioner [GP], nurse, pharmacist prescriber, supplementary prescriber or a hospital doctor in England, through the NHS [National Health Service]). Generally, special LP foods are prescribed by the primary care GP and not by the metabolic centre or metabolic dietitian/clinician. They cannot be purchased over the counter in pharmacists or retail shops.

LP products are essential in the diets of patients with inherited metabolic disorders (IMD) as they: 1) provide a source of energy to support growth and prevent catabolism which may lead to metabolic instability; 2) provide bulk to aid satiety 3) help improve adherence by helping limit the consumption of higher protein foods; and 4) increase the variety of foods consumed. Most GP's only issue LP prescriptions monthly, meaning that the requirement for LP foods must be anticipated in advance. It is commonly a careful balance between ensuring that patients have enough LP food supply to ensure that energy requirements are met but not too much so that it is associated with product ‘stock piling,’ which could lead to wastage and misuse of NHS resources.

Up until 2010 the usual method for obtaining LP products was patient prompted prescriptions that were generated by a GP and dispensed by a community pharmacist. This system was problematic as errors in both prescribing and dispensing of products as well as delays in obtaining products appeared common. Although normal timing for requesting each food prescription is monthly, delays >4 weeks occurred if pharmacies were unable to access LP food supplies. Some patients received incorrect protein substitutes or gluten-free foods instead of LP foods [3]. These errors resulted in distress for patients and their families and placed an additional burden on limited dietetic resources in order to resolve problems.

Following the success of home delivery services for protein substitutes for patients with inherited metabolic disorders (IMD) [3], this service was extended to include distribution of special LP dietary products such as LP bread and pasta. LP dietary foods were delivered by two home delivery companies 1) Homeward® a company which distributed protein substitutes and approximately one third of the UK special LP foods (Loprofin® and Juvela® brands) and 2) Vitaflo at Home® who delivered a small range of LP foods (Fate® and Vitaflo® brands) in addition to protein substitutes produced by Vitaflo®.

It was unclear how effective and efficient the different systems were in terms of ordering patterns, dispensing errors and delays in accessing and delivery of LP foods compared with conventional pharmacy systems. Therefore, we conducted a prospective, longitudinal study, using a questionnaire to examine prescription issues over one year in a group of patients with IMD requiring a LP diet and in receipt of home delivery to access special LP products. We also checked patient's stock levels of special LP foods as well as home storage space and LP food wastage.

## 2 Methods

### 2.1 Patients

Eighty IMD patients requiring a LP diet aged between 0 and 16y were invited to participate in this study. All used the home delivery service for either protein substitute or LP foods. The inclusion criteria to participate in this study were: patients with an IMD disorder of protein metabolism, on a LP diet and using LP foods received by home delivery. Exclusion criteria included: non-use of LP foods and not using at least one home delivery service.

### 2.2 Project design

**Fig. 1:**
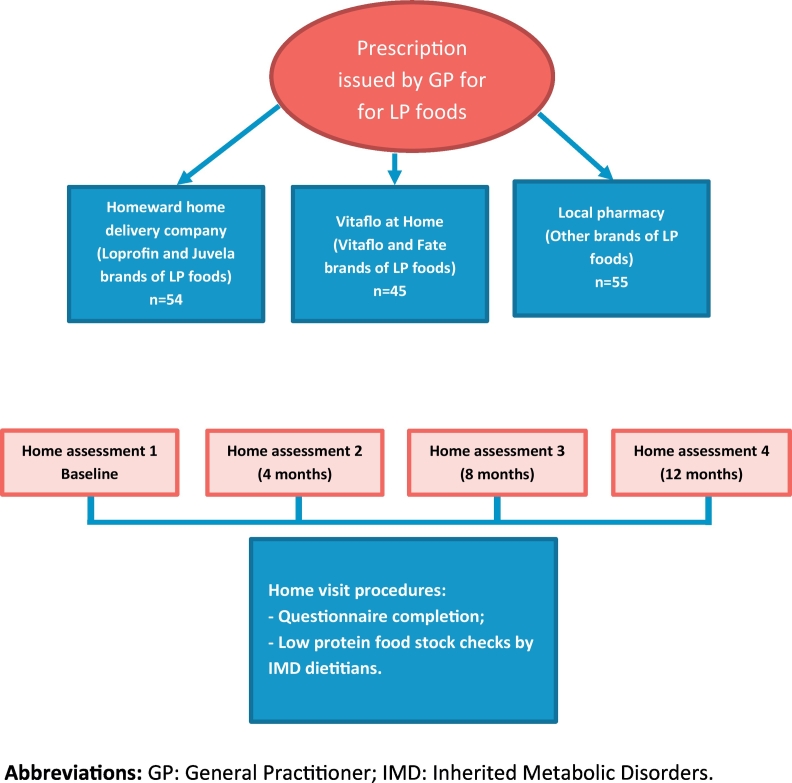
Project design.

For 12 months, home visits where performed by dietitians from the IMD dietetics team as presented by Fig. 1. At each home visit, caregivers were asked to complete a questionnaire consisting of 20 multiple choice and open questions about their prescription experience with special LP foods. The questionnaire was administered on 4 separate occasions over a 12-month period at baseline, 4 months, 8 months and 12 months. It included questions about Homeward®, the local pharmacist and Vitaflo at Home®. Information was collected about the numbers of LP special foods accessed from each source, issues with GP prescriptions of LP foods and any delayed deliveries.

At each home visit, the IMD dietetic researcher also performed a stock check of all LP items stored in the home, examining ‘*use by dates’* and assessed adequacy of storage space for LP items.

### 2.3 Procedures for accessing LP foods

The method of accessing LP items was different for each company. Homeward® requested patients/caregivers to complete a monthly order form listing the LP products required which was returned to the home delivery administrative team. For Vitaflo at Home® a monthly standing order for LP foods was organised for each patient with Vitaflo at Home®. Both companies contacted the GP practices for prescriptions but first established supplies by conducting a stock check with the patients/caregivers. The prescription was then ‘picked’ and verified by a qualified pharmacist and delivered directly to the patient's home. Neither of the home delivery services stocked nor delivered gluten-free foods to avoid delivery error. Any other brands of special LP foods from Promin and PK foods were supplied by the local pharmacist. Caregivers/patients requested prescriptions from the GP which were then delivered to the pharmacist to supply.

### 2.4 Statistical analysis

Data were analysed using descriptive statistics only.

## 3 Results

### 3.1 Patient demographics

In total, 58 patients participated in this study. Forty-nine patients were European Caucasian origin and 9 from Asian origin. Six were unable to speak English as their first language. The diagnosis of patients was: PKU, *n* = 48; tyrosinaemia type I, *n* = 3; tyrosinaemia Type II, *n* = 1; non-pyridoxine responsive homocystinuria, *n* = 2; glutaric aciduria Type I, n = 2; maple syrup urine disease, n = 1; and 3-hydroxy-3- methyl glutaric aciduria, *n* = 1. Fifty seven of 58 patients (98%) were prescribed a protein substitute. All were prescribed special LP foods and already used a home delivery service for protein substitute or LP milk supplies. All patients lived in the West Midland region of the UK.

### 3.2 Questionnaire results

For the delivery of LP foods, 93% (*n* = 54/58) of caregivers used Homeward®, 78% (*n* = 45/58) used Vitaflo at Home® whilst 95% (*n* = 55/58) used their local pharmacy for some LP products unavailable by either home delivery service. Two home delivery services were used by 41 (71%) caregivers and the remaining 17 (29%) only used one of the home delivery service companies. Caregivers obtained most of their LP foods (definition >5 items) from Homeward® (62%, *n* = 36/58) or the local pharmacy (17%, *n* = 10/58). Caregivers were asked about ease of access to their LP products; 83% (*n* = 48/58) said it was easy using Homeward®, compared with 76% (*n* = 44/58) for Vitaflo at Home®, and 57% (*n* = 33/58) their pharmacy. Eighty-one per cent (*n* = 47/58) preferred Homeward® overall.

The number of caregivers reporting the receipt of at least one incorrect prescription for LP foods (e.g. gluten-free foods instead of LP, incorrect product or incorrect amount) was: for pharmacies, 30% (*n* = 16/53), for Homeward®, 6% (*n* = 3/53) and for Vitaflo at Home®, 2% (n = 1/48). Prescription problems with the home delivery services were usually related to items being ‘out of stock’. Forty-nine per cent (*n* = 26/53) said they experienced delayed receipt of LP foods from their pharmacy, compared with 11% (*n* = 6/55) using Homeward® and 8% (*n* = 4/48) from Vitaflo at Home®.

Overall, 45% (*n* = 26/58) reported some difficulty in obtaining prescriptions for LP items from their primary care GP service. Issues included: inability to access all LP products requested (17%, *n* = 10/58), unjustified refusal to prescribe the quantity of LP products requested (19%, *n* = 11/58), prescriptions posted to the incorrect home delivery company/pharmacy (5%, *n* = 3/58), delayed receipt of prescriptions (7%, *n* = 4/58), and caregiver need to ‘track’ delayed prescriptions (12% *n* = 7/58). Forty-three per cent (*n* = 25/58) said they needed more LP foods than they had been prescribed.

### 3.3 Low protein food stock checks

The amounts of special LP food items stored in each household with a child on a LP diet was moderate. The median number of different LP foods stored by each patient was 6 (range 0–22). Households were expected to store adequate food supplies for around 4 weeks. In households, the average amount of special LP food stock held for 4 weeks was a mean of 5 packets of LP pasta, 4 pizza bases, 6 packets LP sausage/burger mix and 3 packets of biscuits (Table 1). This was equivalent to weekly amounts of 1.25 packets of LP pasta, 1.5 packets of LP sausage/burger mix, 1 pizza base, and <1 packet of biscuits. Stocks of LP bread and flour kept by households were minimal.

**Table 1:** Monthly stock levels of low protein food for 58 patients: mean over 12 months.

	Homeward®	Vitaflo at Home®	Chemist
Low protein pasta	5 packets	n/a	<1 packet
Low protein rice	<1 packet	n/a	<1 packet
Low protein bread/bread rolls	< 1 loaf/packet bread rolls	n/a	<1 packet
Low protein pizza base	4 pizza base	n/a	None
Low protein flour	<1 packet	2 packets	None
Low protein burger/sausage mixes	n/a	n/a	6 packets
Low protein breakfast cereals	<1 packet	n/a	<1 packet
Low protein cake mix	n/a	1 packet	None
Low protein crackers	<1 packet	<1 packet	None
Low protein cakes	n/a	n/a	<1 packet
Low protein biscuits	3 packets	n/a	None
Low protein desserts	1 packet	n/a	2 packets
Low protein snack pots	n/a	n/a	<2 pots
Low protein chocolate	n/a	<1 packet	None
Miscellaneous e.g. low protein chocolate spread/snacks	n/a	n/a	<3 units

Forty per cent (*n* = 23/58) of caregivers were assessed to have inadequate storage space for LP foods (i.e. small kitchen with minimal storage cupboards or kitchen with damp conditions). These households were unable to take larger stocks of LP items.

Families discarded minimal LP foods over the 12-month period because of ‘out of date’ shelf life (Table 2). Only 5 of 58 caregivers (*n* = 9%) were identified as keeping higher levels of stock than necessary, with their supplies coming from both the chemist and home delivery service. However, this was usually associated with poor literacy of parents or misunderstanding and over prescription of food items by the GP prescription service.

**Table 2:** Mean wastage of any stock items for 58 patients: mean over 12 months.

	Mean amount
Low protein pasta	0.1 packet
Low protein bread/bread rolls	0
Low protein pizza base	0
Low protein flour	0
Low protein burger/sausage mixes	0
Low protein breakfast cereals	0.1 packet
Low protein cake mix	0.3 pack
Low protein crackers	0.1 packet
Low protein cakes	0
Low protein biscuits	0.5 packet
Low protein desserts	0
Low protein chocolate	0
Miscellaneous	0.1 unit

## 4 Discussion

This study indicated that the number and variety of LP food items ordered and stored by patients and their caregivers is controlled and restrained. It also suggested that caregivers would prefer home delivery services to access their special LP foods although there is no single home delivery service that will supply all LP foods. Unfortunately, whilst access to LP foods is available from pharmacies and more than one home delivery company, with each company only delivering specific brands of special LP foods using different procedures, the overall service is open to error and inefficiencies. Prescriptions are sent to the wrong service and patients and health professionals are confused by the variable paperwork requirements associated with different companies. Generally, for professionals it is a time consuming and fragmented process. Since the completion of this study, another home delivery company has commenced delivery of Promin®, Taranis® and Mevalia® brands of special LP foods, adding to the complexity.

Overall, home delivery services were associated with a lower rate of prescription error and less delay in receiving prescription items. This is similar to the results from a controlled study examining the delivery of amino acid supplements in IMD [3]. In our latest study, we prospectively examined the service over 12 months in order to monitor service fluctuations throughout the year. It was established that home delivery teams were able to consistently identify and correct inaccuracies with prescriptions received from GPs, providing a safer and reliable service. Also, the caregivers preferred home delivery services as it minimised their interactions with primary care health professionals who knew very little about the importance and need for a continuous supply of special LP foods.

In the UK, around 10,500 surgeries deliver primary care services to the UK population. Neither the GP nor their administrative team are likely to have received any specific training in IMD or specialist dietary products. Although IMD dietitians have expert knowledge, and calculate and monitor the complex dietary regimens, they are unable to prescribe special LP foods for their patients; their role is to advise primary care services only. Furthermore, England's NHS budget for LP foods is based within primary care rather than specialist commissioning services (which finance most of the English IMD services). This means that IMD dietitians have little control over the type and quantity of dietary products that are prescribed for their patients. For LP food access to improve, it is essential IMD dietitians are given greater autonomy and accountability for their prescription.

Specialist LP foods are essential to provide adequate energy (around 50% of energy requirements) and satiety in a LP diet and patients should not be left without supplies. This study indicated that some of the patients kept very little reserve stock, suggesting that home delivery services for LP foods were not leading to stockpiling, which has been a concern of primary care services when sending prescription requests to the delivery services. However, the low stock levels of special LP food items held by patients is a concern, particularly the limited amounts of LP bread and flour held, as access to ‘regular’ food supplies using ‘usual’ commercial outlets is not an option. Many caregivers/patients were acutely aware about the cost of treatment and ordered minimal stock which led to low levels of LP food wastage. In fact, some caregivers ordered less food than they needed as they were frightened that their prescription requests may be challenged by the GP services. Others chose to order the same foods each month and not increase variety in order to avoid any criticism by GP practices.

**Fig. 2:**
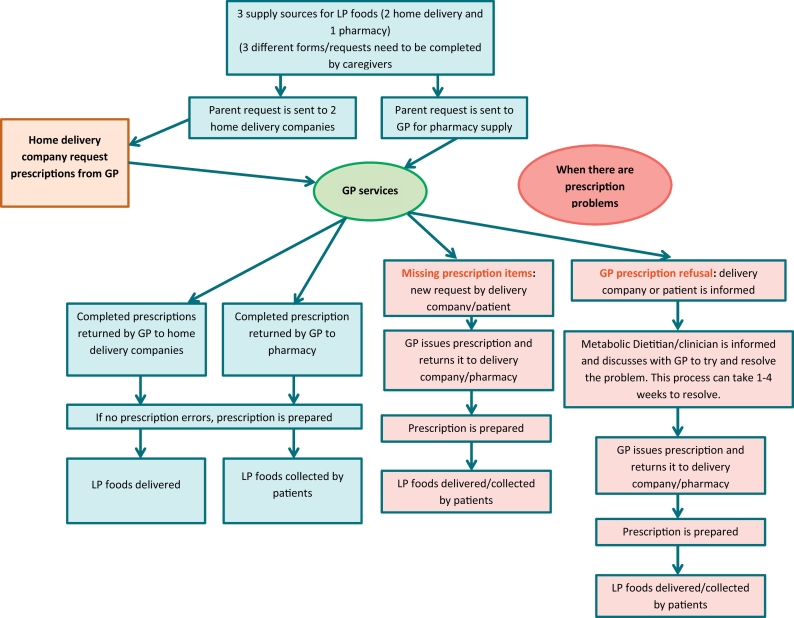
Current system for home delivery/pharmacy service of low protein foods in the UK.

**Fig. 3:**
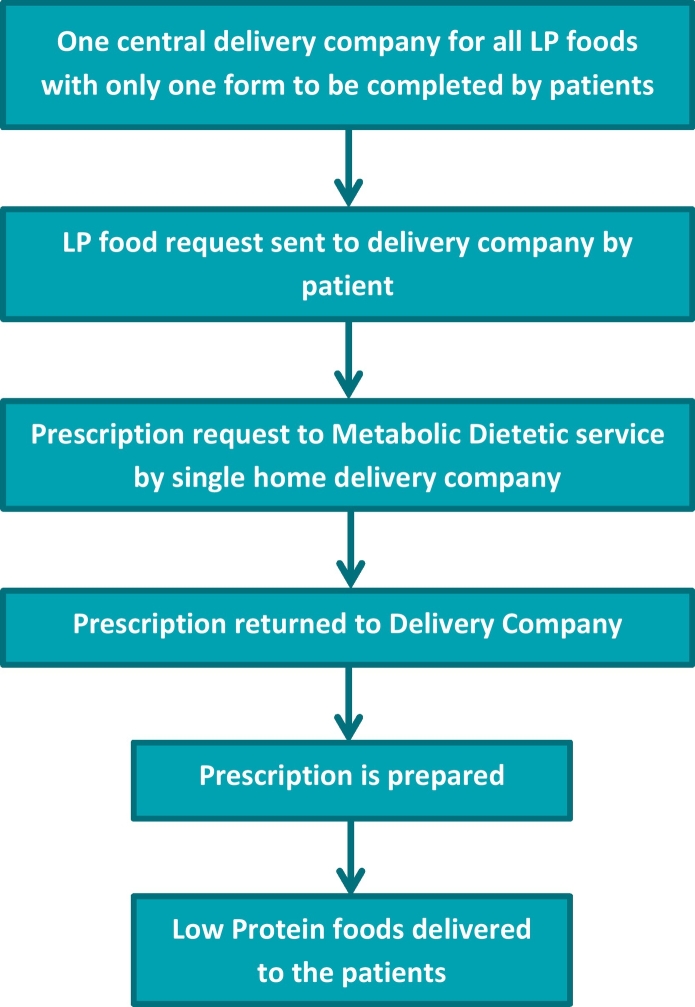
Proposed system for home delivery service of Low protein foods in the UK.

The current system of prescribing LP foods is 47 years old and it is very complex, fragmented, and time consuming (Fig. 2). We believe it is time to reappraise the prescription system and access to LP foods in the UK. We suggest a simpler system based on metabolic dietitians prescribing specialist LP foods using only one centralized delivery company to deliver all specialist LP foods produced by different companies (Fig. 3). We consider that this system would always enable appropriate access to suitable LP foods, and this would be controlled and monitored by specialists such as metabolic dietitians who have knowledge about the patient's specific dietary requirements. NHS resources are likely to be used more efficiently with probable cost savings and patient needs should be better met. This would be fairer for families, IMD teams and GP services. Patients and caregivers should not have to wait 4 weeks for delivery of basic LP foods which are essential for their everyday energy and nutritional needs and metabolic control.

Some European countries use a central distribution point to store and supply all the specialist LP products [2]. Other countries have different national systems such as a government supported financial allocation to patients for their specialist LP foods with patients accessing their own LP foods from web-based LP shops [4]. Some countries expect individual patients to purchase specialist LP foods without financial support, even though they are an essential part of dietary management and cost around 11% of the dietary treatment costs for patients with PKU [5]. It is important to conduct an international multicentre project examining access to specialist LP foods, in order to identify the range of provision, the benefits and pitfalls of each system, the perspective of health professionals and opinions of patients and caregivers. Efficient and effective access to specialist LP foods may be a low priority by health services in some countries, but these foods are an essential component of successful therapy with many inborn errors of protein metabolism. It is important to identify a system of supply which would be a model for other countries to follow.

There are limitations to this study. This was an observational study and we did not include a control group of patients receiving all their LP products via their local pharmacist only. We did not examine dietary patterns of patients or the impact special LP foods have on energy intake. We also did not examine in detail the reasons why a small number of patients had higher amounts of LP food stocks stored in their home. In addition, the questionnaire was not piloted before being used.

## 5 Conclusions

We conclude that the home delivery of special LP foods is associated with less prescription error and is preferred by caregivers and patients. However, prescriptions are issued and controlled by community GP teams who receive no training in rare IMD conditions which is problematic. The access to special LP foods from different sources is also fragmented, confusing and takes considerable caregiver, patient and health professional time to ensure that patients always have access to the correct LP supplies. We believe it is time to adopt a new system based on metabolic dietitians prescribing LP foods with only one UK centralized point delivering all the LP foods. This would be a much simpler, improved, and efficient system, whereby patients would receive all supplies from a single source with minimal burden for patients, caregivers and health professionals.

## Author's roles

All authors participated in data collection, critical revision of the paper and final approval of the version to be published. Anita MacDonald was involved in questionnaire development, data analysis and writing of the manuscript.

## Source of funding

This study was not funded.

## Conflict of interest

Anita MacDonald has received research funding and honoraria from Nutricia, Vitaflo International and Merck Serono. She is a member of the European Nutrition Expert Panel (Biomarin), member of Sapropterin Advisory Board (Biomarin), member of the Advisory Board entitled ELEMENT (Danone-Nutricia), and member of an Advisory Board for Arla and Applied Pharma Research. Alex Pinto has received an educational grant from Cambrooke Therapeutics and grants from Vitaflo, Nutricia, Merck Serono and Biomarin to attend scientific meetings. Sharon Evans is a research dietitian funded by Nutricia; financial support from Nutricia and Vitaflo to attend study days and conferences. Anne Daly has undertaken evaluation work for the nutritional companies – Vitaflo Ltd., Nutricia Ltd. and Metax.

## References

[1] Dixon M.M.A., White F., Stafford J., S V. (2015). Disorders of amino acid metabolism, organic acidaemias and urea cycle disorders. Clinical Paediatrics Dietetics.

[2] Pena M.J., Almeida M.F., van Dam E., Ahring K., Belanger-Quintana A., Dokoupil K. (2015). Special low protein foods for phenylketonuria: availability in Europe and an examination of their nutritional profile. Orphanet J. Rare Dis..

[3] MacDonald A., Manji N., Evans S., Davies P., Daly A., Hendriksz C. (2006). Home delivery of dietary products in inherited metabolic disorders reduces prescription and dispensing errors. J. Hum. Nutr. Diet..

[4] Ahring K.J., Nielsen C.M., Nielsen K.E. (2007). JB. Online ordering of amino acid supplements. J. Inherit. Metab. Dis..

[5] Guest J.F., Bai J.J., Taylor R.R., Sladkevicius E., Lee P.J., Lachmann R.H. (2013). Costs and outcomes over 36 years of patients with phenylketonuria who do and do not remain on a phenylalanine-restricted diet. J. Intellect. Disabil. Res..

